# Introductory Lecture in the Buffalo Medical College

**Published:** 1871-10

**Authors:** 


### Introductory Lecture in the Buffalo Medical College.

Prof. H. N. Eastman, will give the opening Lecture of the course in the College Amphitheatre, Wednesday evening, November 1st, at 7 1-2 o'clock. The lecture will be of interest to the intelligent public, and all are cordially invited to attend.

